# Anesthetic Management of the Pregnant Patient Undergoing Non-Obstetric Surgery

**DOI:** 10.3390/medicina61040698

**Published:** 2025-04-10

**Authors:** Genevieve Monanian, Seth Greenspan, Nadir Khan Yusufzai, Bahaa Daoud, Zhaosheng Jin, Morgane Factor

**Affiliations:** 1 Department of Anesthesiology, Stony Brook University Hospital, Stony Brook, NY 11794, USA; genevieve.monanian@stonybrookmedicine.edu (G.M.); seth.greenspan@stonybrookmedicine.edu (S.G.); bahaa.daoud@stonybrookmedicine.edu (B.D.); morgane.factor@stonybrookmedicine.edu (M.F.); 2 Renaissance School of Medicine, Stony Brook University, Stony Brook, NY 11794, USA; nadiramin.khanyusufzai@stonybrookmedicine.edu

**Keywords:** anesthesiology, pharmacology, physiology, pregnancy

## Abstract

Anesthetic management of the pregnant patient undergoing non-obstetric surgery requires careful consideration of both maternal and fetal well-being. Key factors include appropriate drug selection to minimize fetal exposure, maintenance of uteroplacental perfusion, and management of physiological changes associated with pregnancy, such as altered respiratory function and increased blood volume. Regional anesthesia is often preferred to reduce the risks of general anesthesia, although considerations such as positioning, airway management, and monitoring are crucial. Multidisciplinary collaboration is essential to optimize outcomes, ensuring that both maternal health and fetal safety are prioritized throughout the perioperative period.

## 1. Introduction

Non-obstetric surgery is historically estimated to occur in up to 2% of pregnancies [1]. Although more recent studies cite lower incidences of 0.5–1% of pregnancies [2,3]. Surgery during pregnancy poses unique risks to the fetus as well as to maternal health due to the physiological changes of pregnancy. In consideration of these risks, a committee opinion from the American College of Obstetrics and Gynecology (ACOG) developed along with the American Society of Anesthesiologists (ASA) recommends delaying elective surgery until after delivery, although it emphasizes that pregnant women should never be denied urgent medically necessary surgery [4]. Therefore, the most common surgical procedures during pregnancy are generally non-elective including gastrointestinal (42%), followed by non-obstetric gynecological (20%), and orthopedic or trauma surgery (12%) [3].

Many clinical concerns arise when providing anesthesia for non-obstetric surgery during pregnancy. The benefits and risks of neuraxial or regional anesthesia versus general anesthesia must be weighed carefully, in addition to the technical challenges of neuraxial anesthesia in this population [5]. Additionally, the physiologic changes of pregnancy provide concerns for increased risk of pulmonary aspiration, coagulopathy, and different pharmacological distribution and metabolism of anesthetic drugs. The pregnancy state itself produces clinical concerns of inducing pre-term labor, potential teratogenicity of anesthetic drugs, and long-term risk to the health of the fetus or the mother. This narrative review will discuss the unique challenges to the anesthesiologist when caring for pregnant patients undergoing non-obstetric surgery.

## 2. Search Strategy

The authors searched the databases PubMed, Embase, and Google Scholar for relevant articles to this review. The following search terms and their combinations were utilized: pregnancy, non-obstetric surgery, general anesthesia, pre-term labor, teratogenicity, prenatal exposure, pregnancy complications, pregnancy pathophysiology, pregnancy complications, pregnancy physiology, anesthetic management in pregnancy, pulmonary aspiration in pregnancy, airway management in pregnancy, mechanical ventilation in pregnancy. Article types considered for screening included review articles, basic science reports, clinical trials, observational studies, reports/series, meta-analyses, and systematic reviews. The references were screened by authors and selected for inclusion based on relevance to the topic and quality of evidence. The resulting body of evidence was synthesized by authors into this narrative review article which describes key anesthetic considerations for providing anesthesia for non-obstetric surgery in pregnancy.

## 3. Maternal Physiological Changes

**Figure 1:**
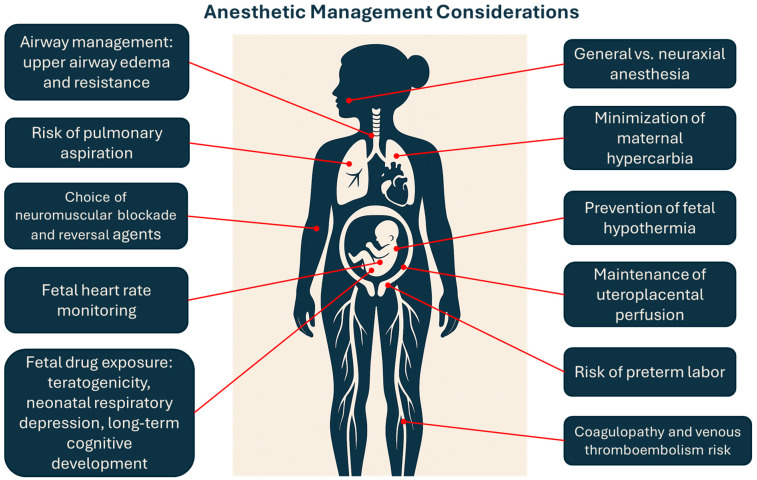
Summary of anesthetic considerations during pregnancy.

The metabolic demands of pregnancy require several physiological changes, influencing nearly all organ systems. These changes are determined by hormonal and mechanical factors. The effects of pregnancy on the cardiovascular, respiratory, hematologic, and gastrointestinal systems are discussed here (Figure 1).

### 3.1. Cardiovascular

Pregnancy induces notable physiological changes in the cardiovascular system. Cardiac output increases by 20% to 45%. It rises non-linearly during early pregnancy, peaking by the early third trimester in singleton pregnancies before marginally declining toward term. Early increases in cardiac output are more pronounced in multiple gestations [6]. In early pregnancy, the heart rate begins to rise, reaching its peak in the third trimester. The extent of the increase varies between individuals, but it has been shown to rise by 10 to 20 beats per minute by the end of pregnancy, which often represents a 20% to 25% increase in resting heart rate [7].

During the first trimester, systemic vascular resistance, or afterload, decreases, prompting the activation of the sympathetic nervous system and thus an elevated heart rate. The stroke volume increases by 20% to 30% while the plasma volume increases by 30% to 50% [8].

Plasma volume increases to meet the metabolic demands of the placenta and fetus. An increase in plasma volume becomes noticeable at 6–8 weeks. This increase peaks at 32 weeks. Plasma volume may exceed 3.5 L at 38 weeks of gestation, complementing increases in total body water in all body fluid compartments [9]. Increases in blood volume and cardiac output can lead to decompensation in pre-existing structural cardiac conditions, such as valvular lesions [10].

Systolic blood pressure is typically unchanged while diastolic blood pressure decreases by approximately 10 mmHg. Diastolic blood pressure approaches its lowest point at 24–26 weeks before rising again. Fluctuations in blood pressure are common and are influenced by maternal positioning and systemic vascular resistance, which decreases because of the effects of estrogen, progesterone, nitric oxide, and endothelin [8]. Decreases in blood pressure and systemic vascular resistance lead to an increased risk of hypotension following anesthesia [10]. Hypotension caused by spinal anesthesia particularly causes the dilation of large veins, such as the vena cava, which reduces blood return to the maternal heart, subsequently lowering the supply of oxygenated blood to the fetus. Maternal hypotension induced by spinal anesthesia during cesarean delivery is a recognized issue that can impact both the mother and fetus [11].

### 3.2. Respiratory

During pregnancy, the respiratory rate remains unchanged. Minute ventilation increases by 30% to 50%, primarily because of an increase in tidal volume, which can increase by 40%. Changes in minute ventilation typically occur early in pregnancy during the first trimester and remain stable or increase slightly as pregnancy progresses [12]. Oxygen consumption is increased by 20% during pregnancy. This consumption is adjusted by an increase in cardiac output [8,13].

In nonpregnant women, tidal volume depends on diaphragmatic movement, while in pregnant women, it is influenced by both diaphragmatic movement and intercostal muscle activity. The hyperventilation that occurs during pregnancy is due to elevated progesterone levels, increased metabolic demands, and increased CO_2_ production. Progesterone enhances sensitivity to CO_2_ levels, stimulating brain regions that regulate ventilation such as the hypothalamus, thalamus, and medulla oblongata [13]. A state of compensated respiratory alkalosis develops due to progesterone-driven hyperventilation. Arterial pH levels are slightly elevated (7.40–7.45) [14]. Progesterone levels gradually rise throughout pregnancy, starting at 25 ng/mL at 6 weeks and reaching 150 ng/mL by 37 weeks of gestation [15].

The diffusion capacity for carbon monoxide peaks during the first trimester. It subsequently decreases to its lowest average value between 24 to 27 weeks and then rises again postpartum [13]. Pregnancy is characterized by factors that typically increase airway resistance, such as upper airway edema and reduced FRC, which decreases by 20%. However, airflow rates remain stable throughout pregnancy, likely because of hormonally mediated bronchodilation effects. The elevation of the diaphragm, reduced outward recoil of the chest wall, and decreased downward tension of the abdomen resulting in the decreased FRC observed during pregnancy [16]. This reduction is worsened by the supine position. Expiratory reserve volume decreases by approximately 200 mL, while residual volume demonstrates a small or nonsignificant decrease [13]. This decrease in the expiratory reserve volume occurs during the second half of pregnancy, with a reduction of 8-40% by term [15]. Total lung capacity remains unchanged, however, as an increase in inspiratory volume counterbalances the decrease in FRC.

Because the gravid uterus elevates the diaphragm in pregnant patients, low tidal ventilation strategies are recommended in cases of respiratory failure, as they prevent barotrauma while maintaining adequate oxygenation. Positive end-expiratory pressure enhances oxygenation and should be utilized to maintain a PaO_2_ > 65 mmHg while minimizing FiO_2_. The target PaCO_2_ is 30–32 mmHg, as this reflects normal physiological levels during pregnancy. Significant respiratory alkalosis should be avoided, as it may reduce uterine blood flow. Conversely, maternal permissive hypercapnia can lead to fetal respiratory acidosis, which may be harmful, though small trials have demonstrated its safe use in select pregnant patients [17].

Pregnancy causes histologic changes in the nasopharyngeal and oropharyngeal mucosa, including increased activity and secretions. These upper airway changes contribute to the development of sleep-disordered breathing and a reduction in the oropharyngeal junction [10,13]. Pregnancy rhinitis, which leads to nasal congestion, affects up to 42% of women by the third trimester, without any known allergic reaction. The frequency of sleep-disordered breathing symptoms such as snoring, breathing pauses, and choking significantly increases from the first trimester to the month of delivery. Pregnancy may also be associated with increased daytime sleepiness [18].

Thus, the physiological changes that occur during pregnancy are associated with difficulties in airway management. The edema and vascularity of the upper airway raise the risk of swelling and bleeding, which can occur rapidly and worsen during labor [13].

Moreover, the metabolic rate in pregnant women increases to meet the demands of the fetoplacental unit. An imbalance between oxygen delivery and consumption elucidates why pregnant women may experience accelerated desaturation following the induction of general anesthesia. Careful preoxygenation before procedures, especially under general anesthesia, is essential to ensure adequate oxygen reserves [8]. Pregnant women have significantly reduced safe apnea times. Moreover, the risk of stomach content aspiration increases due to decreased lower esophageal sphincter tone [19]. This risk is caused by the actions of progesterone and the upward displacement of the stomach and diaphragm.

### 3.3. Hematologic

The physiological changes of pregnancy increase the risk of hypercoagulability, making it 5–10 times higher than in nonpregnant individuals [20]. This increased risk remains until approximately 6–12 weeks postpartum [21]. The risk of thrombosis, though low, remains elevated for up to six months after childbirth before gradually returning to baseline levels. This hypercoagulability stems from altered levels and activity of clotting factors. Factors VIII, IX, and X are elevated, and fibrinogen production increases by up to 50%. Moreover, fibrinolytic activity, protein S concentrations, and antithrombin concentrations decrease during pregnancy. The functional activities of protein S and antithrombin also decrease, shifting the hemostatic balance towards thrombosis [22]. These changes affect all three factors of Virchow’s triad, which consist of endothelial dysfunction or injury, hemodynamic alterations, and hypercoagulability.

Most of the increase in plasma volume that develops during pregnancy occurs between 32 to 34 weeks of gestation [23]. Rises in plasma volume are proportional to the baby’s birth weight. Hematocrit, red blood cell count, and hemoglobin concentration decrease due to the increase in plasma volume surpassing the increase in red blood cell mass. The mean corpuscular volume and mean corpuscular hemoglobin concentration are generally unaffected by this dilution. The platelet count typically remains within normal limits. Iron requirements increase by two to three times to support fetal development, enzyme production, and hemoglobin synthesis. Folate needs rise by 10 to 20 times, while vitamin B12 requirements double [24].

Pregnancy-induced basal tachycardia and hemodilution can delay the onset of classical signs of hypovolemia, complicating early recognition and management [8]. Additionally, the hypercoagulable state associated with pregnancy necessitates careful perioperative planning, including the use of thromboprophylaxis to reduce the risk of deep vein thrombosis. Pharmacological thromboprophylaxis should be discontinued prior to surgery to ensure the safe administration of neuraxial techniques.

### 3.4. Gastrointestinal

Gastroesophageal reflux is a frequent issue during pregnancy, primarily because of a decrease in gastric secretion pH, an increase in secretion volume, and a decrease in lower esophageal sphincter tone. The decreased tone of the lower esophageal sphincter stems from the actions of progesterone on smooth muscle cells. These actions further contribute to nausea and vomiting, which affect approximately 80% of pregnant individuals [25]. These gastrointestinal symptoms can begin as early as the second week and persist until the second trimester, and in some cases, until 37 weeks’ gestation or full term. These symptoms can alter drug absorption and bioavailability. It is recommended that patients take medications when nausea is minimal to avoid decreased bioavailability, which may occur due to lower drug concentrations at the absorption site [8].

Some studies suggest that increased estrogen and progesterone levels contribute to the relaxation of smooth muscle cells in the gastrointestinal tract, leading to delays in gastric emptying [26]. These delays may result in constipation and bloating, and worsen nausea and vomiting. However, some studies report no delay in motility throughout pregnancy [27]. Thus, there is ongoing debate about whether gastric emptying and motility are delayed during pregnancy [8].

### 3.5. Uteroplacental Circulation

As pregnancy progresses, uteroplacental blood flow increases to ensure a sufficient supply of oxygen and nutrients, which is vital for maternal health and fetal growth and development [28]. This section explores the dynamics of blood flow to the uterus and placenta, along with the factors influencing drug transfer across the placenta.

### 3.6. Blood Flow

Uteroplacental circulation, which connects maternal and fetal blood supplies, is established early in the second trimester [29]. During pregnancy, uteroplacental vessels adapt to support an increased blood flow demand. Evidence from human and animal studies demonstrates that this adaptation includes functional changes in vascular smooth muscle cells and endothelial cells, such as the remodeling of spiral arteries. The remodeling of spiral arteries and the functional adaptation of uterine arteries transform this circulation into a low-resistance, high-flow system [29,30]. Adequate uteroplacental blood flow is essential for both fetal growth and maternal health. Impaired uteroplacental vascular transformation or adaptation is linked to pregnancy complications such as preeclampsia and fetal growth restriction.

Unlike most organs, where flow resistance primarily occurs at the innervated arteriolar level, the placenta lacks direct innervation. However, the vascular smooth muscle tone in the placenta responds to local transmural pressure and vasoactive substances [31]. These regulatory substances include hormones transported via the bloodstream, such as angiotensin II and atrial natriuretic peptide, neurotransmitters released from nerve endings, such as norepinephrine and acetylcholine, endothelial-derived factors, such as nitric oxide and prostaglandins, hormones from endocrine tissues, such as estrogens and progestins, and molecules released from surrounding vascular cells [30].

### 3.7. Determinants of Placental Transfer

A significant concern regarding drug use during pregnancy is the transfer of medications across the placental barrier, which can result in fetal drug exposure and potential toxicity to the developing fetus. 5–10% of pregnant women are prescribed drugs with potential teratogenic effects, and around 50% of these medications are administered during the first trimester, a critical period for fetal development and increased susceptibility to toxins [32]. While most drugs can cross the placenta to some extent via passive diffusion, the placenta also contains numerous adenosine triphosphate binding cassette efflux transporters and solute carrier uptake transporters [33]. These drug transporters play an essential role in regulating fetal drug exposure. They are expressed in the synctiotrophoblast cell layer of the placenta, which separates fetal and maternal circulations [34].

Lipophilic, non-ionized small molecule drugs can pass through the placental barrier via passive diffusion. Passive diffusion rates are influenced by factors such as the concentration gradient across the placental barrier, surface area, placenta thickness, and the molecular weight and lipophilicity of the drug [35]. The binding of the drug to maternal and fetal plasma proteins, the ionization of the drug in maternal and fetal blood, and the rate of maternal blood flow to the placenta can impact the diffusion process as well. Conversely, the transfer of hydrophilic drugs with low membrane permeability requires facilitated or active transport, mediated by transporter proteins expressed in the placenta. The efficiency of transporter-mediated drug transfer across the placenta is determined by the transporters’ intrinsic activity for the drug and its abundance in the placental tissue.

### 3.8. Drug Metabolism and Pharmacokinetics

Physiological changes during pregnancy influence the bioavailability, distribution, and clearance of various drugs. During labor, gastric emptying is slowed due to factors such as pain, anxiety, and opioid use, which delays drug absorption in the intestines. Increases in maternal weight and intravascular fluid volume expand the volume of distribution for water-soluble drugs. Increased renal blood flow and glomerular filtration rates increase the clearance of drugs that are eliminated through the kidneys. Given the combined effects of increased volume and clearance during pregnancy, changes in drug half-life cannot be easily predicted, requiring individual evaluation of each drug [36].

With regards to drugs frequently utilized in anesthetic practice, propofol is commonly used for the induction of general anesthesia in sedative-hypnotics. However, the pharmacokinetic and pharmacodynamic changes of propofol during pregnancy are not well understood [34]. Neuromuscular blocking agents such as rocuronium are affected by the physiological changes described above. Furthermore, increases in plasma volume lead to a decrease in plasma cholinesterase levels. This impacts hepatic clearance rates and leads to variations in the onset and duration of action of neuromuscular blocking agents. As a result, it is crucial to closely monitor neuromuscular function to avoid a rapid rise in circulating acetylcholine, which could trigger uterine contractions [37]. The clearance of intravenously administered opioids such as morphine increases by 60–70% during pregnancy compared to the postpartum period. Because morphine is a high-extraction-ratio drug, its clearance is dependent on changes in hepatic blood flow. Thus, its increased clearance occurs because of increased hepatic blood flow in pregnancy [38].

## 4. Key Factors for Consideration

### 4.1. Placental Transfer

When nonobstetric surgery is performed in a viable pregnancy, there is a clinical concern about the placental transfer of anesthetic drugs and subsequent effects on the neonate if there is an unanticipated preterm delivery. Many older studies have examined the placental transfer of general anesthetic drugs and their effect on the neonate in the context of cesarean section. Volatile anesthetics including halothane, sevoflurane, isoflurane, and desflurane have been shown to cross the placenta in relatively equal amounts and are associated with decreased neonatal APGAR scores at one minute but not 5 min suggestive of their short duration of action [39]. Propofol is found in fetal circulation when used for general anesthesia for cesarean section or for surgery earlier in gestation, although umbilical vein propofol concentrations do not correlate with APGAR scores after delivery [40,41]. Dexmedetomidine is widely used in pediatric anesthesiology including infants and has been safely used as an adjunctive medication for cesarean sections without an effect on neonatal APGAR scores or cord blood gas concentrations [42]. Benzodiazepines such as midazolam can cross the placenta and maintain a significant half-life in the neonate after delivery [43]. However, a randomized trial comparing midazolam versus placebo for anxiolysis prior to spinal anesthesia for cesarean section found no difference in neonatal APGAR scores between the midazolam and the control group [44].

Systemic opioids will cross the placenta and place a fetus at risk for neonatal respiratory depression after delivery, although the duration and extent of this sedation will vary by the specific drug. Meperidine has been commonly used for labor analgesia and its effects on the neonate have been well described including respiratory depression and metabolic acidosis which can last hours after delivery depending on the proximity of administration to delivery [45]. Fentanyl, morphine, and alfentanil have also been used for labor analgesia and will similarly pose risks of neonatal respiratory depression [45]. Remifentanil also has notable placental transfer but unlike other opioids is rapidly metabolized in the fetus and has not been shown to affect APGAR scores [46]. Neuraxial anesthesia is the most common anesthetic technique for cesarean delivery, as general anesthesia has been associated with concern for difficult maternal airway management and increased fetal drug exposure [47]. General anesthesia for emergency cesarean section has also been associated with lower Apgar scores and the need for increased neonatal resuscitation and intensive care admission [47,48]. Conversely for neuraxial anesthesia, placental transfer of commonly used long-acting local anesthetics (i.e., bupivacaine, ropivacaine) should be of minimal concern assuming high concentrations have not been inadvertently administered intravenously. Neuraxial opioids are also of lesser clinical concern for neonatal respiratory depression than when administered systemically [45].

Both nondepolarizing and depolarizing neuromuscular blocking drugs are well known to be poor at placental transfer due to their lipophilicity, ionization, and strong protein binding [49]. More clinical concern may exist for reversal agents as acetylcholinesterase inhibitors such as neostigmine are commonly used to reverse paralysis for surgery in pregnant patients. Neostigmine has theoretically limited placental transfer due to its large quaternary structure but can still pose a risk of fetal bradycardia when unopposed by anticholinergic agents. However, it crosses the placenta in a greater ratio than the quaternary amine glycopyrrolate, therefore the tertiary amine atropine is the preferred agent for coadministration with neostigmine [50].

Sugammadex has not been well studied in pregnant patients and concern about potential harm has led most anesthesiologists to prefer the aforementioned traditional acetylcholinesterase inhibitors as reversal agents for nonobstetric surgery in pregnancy. The placental transfer of sugammadex is unknown, although its large size polarization makes a substantial transfer to the fetus unlikely [51]. At the time of writing, there are no known case reports of sugammadex administration shortly before delivery, an expectedly rare clinical scenario.

Regardless of the anesthetic choice, if delivery is anticipated in the perioperative period, the risk of neonatal respiratory depression from general anesthetics or opioids should be considered and an appropriate neonatal resuscitation team should be available.

### 4.2. Teratogenicity

All anesthetic agents have been shown to be teratogenic in at least one animal model at some time point in pregnancy, although none have been definitively shown to be teratogenic in humans [52,53]. Nitrous oxide can inactivate Vitamin B12 which can inhibit the synthesis of methionine, an essential precursor to DNA substrates, leading to hematological and neurological disease in chronic users of recreational nitrous oxide [54]. This phenomenon has led to concern for the inhibition of fetal DNA synthesis if nitrous oxide is administered during the organogenesis of early labor and some authors discourage its use in early pregnancy especially considering the availability of many other safe anesthetics [52]. Interestingly, xenon, an anesthetic gas of increasing interest to researchers, but not yet widely used in clinical practice, has low potential for teratogenicity at least when compared to nitrous oxide in animal studies [55].

Sugammadex deserves special consideration as a novel drug. It has been associated with apoptosis or necrosis of primary neuronal cell cultures of fetal rats [56]. It was also associated with neuronal apoptosis of neonatal mice but only when administered alongside sevoflurane [57]. However, a recent retrospective study found no difference in preterm labor or miscarriage between pregnant patients who underwent general anesthesia with or without sugammadex within four weeks of exposure [58]. Additionally, a case series of six patients who had general anesthesia with exposure to sugammadex reported no adverse events intraoperatively or to the health of the babies after they were born [59].

Local anesthetics are similarly safe to use in pregnancy, except for cocaine which when used recreationally is associated with maternal cardiovascular, renal, and hepatic disease as well as adverse perinatal outcomes including preterm delivery, low birth weight, placental abruption, and intrauterine fetal demise [60]. As mentioned previously, substantial placental transfer of local anesthetics when used for neuraxial anesthesia is unlikely unless inadvertently administered intravascularly [45].

### 4.3. Long-Term Effects

The evidence for long-term health issues in children exposed to anesthesia prenatally or early in childhood is an area of increasing concern and research. The FDA released a statement in 2016 that “repeated or lengthy use of general anesthetic and sedation drugs during surgeries or procedures in children younger than 3 years or in pregnant women during their third trimester may affect the development of children’s brains” [61]. This recommendation was made based on a compositive of evidence from animal studies as well as observational studies and clinical trials of young children. However, to our knowledge, the FDA has not referenced any human studies involving pregnant participants when drafting this guideline. Additionally, the FDA acknowledges the possibility of confounding bias in studies on young children, as the development of neurocognitive disorders may be a consequence of the underlying conditions that led to the surgery rather than the long-term effects of anesthetic drugs [61].

Despite these risks, consistent with the ACOG/ASA recommendations, the FDA continues to recommend that medically necessary surgery not be delayed in pregnant patients. Additionally, the FDA also goes on to state that while the label change applies to anesthetic drugs that antagonize N-methyl-D-aspartate (NMDA) receptors and/or potentiate gamma-aminobutyric acid (GABA) activity, no specific anesthetic drug has been proven to be more harmful than any other. Therefore, the FDA suggests that short single exposures to general anesthesia in the third trimester of pregnancy have not convincingly been associated with long-term harm to children’s neurocognitive development [61].

Several studies published after the FDA guidelines have specifically examined the potential long-term effects of prenatal anesthesia exposure on children’s cognitive development. A prospective study including 22 mothers who were exposed to general anesthesia in pregnancy found that their children were more likely to have worse externalizing behavioral scores on a standardized assessment taken at age 10 when compared to controls without prenatal anesthesia exposure [62]. However, there were no differences found in assessments for other neurocognitive domains including language, cognition, and motor function [62]. The majority of prenatal anesthetic exposures in this study occurred in the first trimester, although the authors did not evaluate for statistical differences based on the trimester of exposure due to the small sample size [62].

However, a prospective cohort study which examined 129 children who were exposed to anesthesia prenatally and who were matched to unexposed children found no behavioral differences on a questionnaire or DSM diagnoses of behavioral disorders between exposed and unexposed children [63]. However, in exploratory analyses, the authors found a statistically significant association between prenatal anesthetic exposure and worse executive function but not psychosocial problem scores among children exposed to general anesthesia, laparoscopic surgery, or greater than 1 h of anesthesia prenatally [63]. A limitation of this study is that it uses parental reports of children’s changes in behavior which limits the ability of the study to detect more minor disorders of neurocognition that do not cause overt behavioral change.

A much larger recent Medicaid database study of 34,271 children exposed to anesthesia prenatally for maternal appendectomy or cholecystectomy who were matched to children without prenatal anesthesia exposure found a 31% increase in diagnosis of behavioral disorders in the exposure group [64]. Associated disorders included speech and language disorders, attention-deficit/hyperactivity disorder, and autism with the greatest increases in risk associated with anesthesia administered during the second and third trimesters [64]. The above studies on prenatal anesthesia exposure are limited by their observational design. They have no methods of elucidating whether it was the anesthesia that was associated with these behavioral outcomes or the surgical pathology that indicated the operation.

### 4.4. Pre-Term Labor

When non-obstetric surgery is performed in a viable pregnancy, there is concern for potential preterm labor or even intrauterine fetal demise. A recent systematic review and meta-analysis including 80,205 women who underwent non-obstetric surgery and observed a variety of outcome measures found that non-obstetric surgery was associated with twice the odds of preterm birth with a prevalence of 9.8% in patients who underwent non-obstetric surgery versus 4.9% in those who had not [3]. The risk of preterm delivery was lower for patients who underwent laparoscopic surgery, suggesting a minimally invasive approach may minimize complications when feasible. For the outcome of intrauterine fetal demise, the reviewers also found increased risk among patients who underwent nonobstetric surgery (prevalence 1.3% versus 0.5%) [3].

A small retrospective study of 77 patients who underwent nonobstetric abdominal surgery in pregnancy found higher rates of preterm labor with appendicitis than with biliary tract pathology, and the authors suggest that inflammation near the uterus may directly increase this risk [65]. Additionally, there were substantially more preterm deliveries in the third trimester (82% of patients) than in the second (26% of patients), suggesting the second trimester is the safest time for surgery during pregnancy if feasible [65]. A meta-analysis evaluating primarily abdominopelvic non-obstetric surgery in pregnant women similarly found a higher incidence of preterm labor associated with pelvic surgery such as appendectomies and adnexal surgery versus upper abdominal surgeries such as cholecystectomy [66].

The risk of preterm labor has led ACOG to recommend fetal heart rate and contraction monitoring before and after non-obstetric surgery in viable pregnancies and to consider intraoperative monitoring if consent is obtained and qualified personnel are available to perform an emergency cesarean delivery [4].

## 5. Anesthetic Management

### 5.1. Preoperative Evaluation

Preoperative evaluation of the pregnant patient undergoing non-obstetric surgery or invasive procedures is one that should be conducted with a multidisciplinary approach. There are several considerations to be given to maternal physiology, fetal physiology, and overall maternal-fetal well-being. Key stakeholders to participate in the care of this patient include the primary surgical team, obstetrics, maternal-fetal medicine if indicated, and anesthesiology. Consultation with an obstetrician is paramount as they are specialists regarding maternal-fetal well-being. Guidelines provided by the American College of Obstetrics and Gynecology state that a pregnant patient should never be denied necessary non-obstetric surgery or have it delayed regardless of trimester [4]. Truly elective surgery should be delayed until the postpartum period.

Prior to personally counseling the patient, it is helpful to remember a few key points related to anesthetic care in the pregnant patient. The first is that there are no currently used anesthetic agents which have been shown to be teratogenic regardless of gestational age. Additionally, there is no evidence that exposure to anesthetic or sedative drugs in utero has any effect on the developing fetal brain and there is no animal data to support an effect on fetal brain development when exposures are less than 3 h in duration [4].

The preoperative evaluation of a pregnant patient by the anesthesiologist should contain the core tenets of a preoperative evaluation of any patient. The basic standards for preanesthetic care as defined by the American Society of Anesthesiologists begin with a review of the available medical record for the patient and then give way to an interview and focused examination of the patient. The goals of this preoperative evaluation are to discuss the patient’s medical history, previous anesthetic experiences, and current medical therapy. A difference for this evaluation in the pregnant patient would also be to elicit the patient’s obstetrical history including gestational age and any complications. With this history obtained the goal is then to assess the qualities of the patient’s physical condition and determine how this may increase the patient’s perioperative risk. In this scenario, the anesthesiologist must consider maternal as well as fetal risk and make decisions to help mitigate that [67]. In regard to the pregnant patient, perioperative considerations and risks that frequently come to mind are aspiration risk, potential for preterm labor, the hypercoagulable state with risk of venous thromboembolism, and the need for preoperative anxiolysis.

Concern for aspiration stems from physiologic changes of pregnancy such as decreased lower esophageal sphincter tone due to progesterone, increased intragastric pressures from the growing uterus, and decreased functional residual capacity leading to rapid desaturation during a period of apnea [68,69]. Given the traditional concern for pulmonary aspiration based on these physiologic changes, the use of rapid sequence intubation (RSI) is frequently implored with some recommending RSI from the 12th week of pregnancy, 13th week of pregnancy, 18th week of pregnancy, and 27th week of pregnancy [70,71,72,73]. However, it is important to note that literature has shown gastric emptying in pregnant patients to only be delayed during active labor and that gastric volumes in pregnant patients are like the non-pregnant patients otherwise [74].

Multiple large studies have failed to identify pregnancy as a risk factor for pulmonary aspiration and the Serious Complications Repository (SCORE) project did not identify any cases of pulmonary aspiration in over 96 k Caesarean deliveries, over 5 k of which were completed under general endotracheal anesthesia [75,76]. Similarly, a retrospective review of pregnant patients with 50 k in the first trimester and 11 k in the second trimester undergoing deep sedation with propofol without endotracheal intubation did not show any cases of pulmonary aspiration [77]. Finally, the closed claims analysis for pulmonary aspiration of gastric contents published in 2021 reported a total of 115 events with only 4 being in pregnant patients, 3 of which occurred during Caesarean section [78]. Therefore, as an anesthesiologist in the preoperative evaluation phase for a pregnant patient undergoing a non-obstetric procedure it is important to consider the recent literature when determining the risk of aspiration for the pregnant patient. In the pregnant patient without obesity who has met the appropriate nil per os guidelines for surgery, the risk of pulmonary aspiration is low and not, unlike a non-pregnant patient. Consideration for RSI and or administration of prophylactic non-particulate antacids should be tailored to patient-specific presentation.

Many pregnant patients undergoing non-obstetric procedures will express concern regarding preterm labor. Again, the involvement of obstetrics during this time is paramount. The gestational age and viability of the fetus are the first factors to examine. A fetus is generally considered viable at 24 weeks gestation with the peri-viable period typically referring to 20 weeks through 25 weeks and 6 days gestation [79]. If the fetus is considered pre-viable then based on organizational guidelines fetal well-being should be confirmed with the use of fetal heart rate detection via Doppler prior to the procedure. If the fetus is considered viable, then simultaneous electronic fetal heart rate and contraction monitoring should be performed in the presence of an obstetrician qualified to interpret it. Finally, obstetricians may consider administering corticosteroids to the pregnant patient for fetal benefits in the setting of a viable preterm gestational-age fetus [4,9].

In terms of quantifying the risk of pregnancy loss or preterm labor, the overall risk of adverse obstetrical events during non-obstetric surgery is low and patients should be counseled as such. Data from a systematic review of more than 12,000 surgeries during pregnancy showed an incidence of 10.5% first-trimester pregnancy loss and an overall miscarriage rate of 5.8%. The baseline risk for miscarriage is approximately 10% of all pregnancies in the first trimester and <1% of all pregnancies in the second trimester [80].

The pregnant patient is at 5–10 times the risk of hypercoagulability when compared with nonpregnant patients [20]. This risk remains elevated even until the postpartum period [21]. In the preoperative phase, the anesthesiologist must take care to discuss perioperative thromboprophylaxis for all patients with the surgical team. Some surgical teams may be unfamiliar with the risk posed to this patient population and care should be taken to prevent venous thromboembolism in the perioperative period. Additionally, one must identify patients who are already on pre-existing anticoagulation and there must be coordination with the surgical team regarding management both for the procedure and potential neuraxial anesthesia.

Finally, the anesthesiologist must evaluate the need for preoperative anxiolysis in the pregnant patient. Typically, preoperative anxiolysis is achieved with the administration of midazolam to the patient. There has been hesitation regarding the administration of benzodiazepine anxiolysis to pregnant patients due to older studies showing a possible link between maternal diazepam use and the development of cleft palate in the fetus. However, more recent and larger studies have failed to demonstrate an association between the two although it could not be excluded [81]. Additionally, it is important to note that midazolam specifically has not been associated with any congenital malformations [82]. Several reviews cite the preference for verbal reassurance instead of pharmacologic anxiolysis. However, recent studies have begun to explore the benefits of decreased stress response and catecholamine release in pregnant patients following preoperative anxiolysis with a benzodiazepine. Therefore, like any preoperative evaluation, careful titration of benzodiazepine premedication with the goal of anxiolysis should be considered in the patient experiencing anxiety and stress in the face of impending surgery [37].

### 5.2. Intra-Operative Management

When transitioning to intraoperative management of the pregnant patient undergoing non-obstetric surgery, one of the first things to consider is the positioning of the patient. After 18–20 weeks of gestation, it is ideal to achieve a position that allows both adequate surgical exposure and avoids aortocaval compression to ensure adequate uteroplacental blood flow. This is typically achieved by positioning with left uterine displacement [23,37]. However, there are instances in which left uterine displacement may not be possible due to surgical exposure needs. It is important to remember that uteroplacental blood flow can still be maintained with adequate maternal systolic blood flow and that in the instance of hypotension, if safe from a surgical perspective, the patient can be placed in left uterine displacement or a fully left lateral position [23].

Monitoring of the pregnant patient should consist of the standard monitors outlined by the American Society of Anesthesiologists. Advanced or invasive monitors would be determined by specific risks from the type of surgery or patient factors. Fetal monitoring during the surgical procedure should be judged by the gestational age and fetal viability. Organizational guidelines recommend that previable fetal well-being should be judged by a preprocedural Doppler of fetal heart rate, whereas fetuses of viable gestational age should have a simultaneous electronic fetal heart rate and uterine contraction monitoring prior to the procedure. Continuous fetal heart monitoring during the procedure can be considered, however, there are caveats. If continuous monitoring is going to be pursued during the procedure it is important that an obstetrician be present to interpret this monitoring, that this obstetrician is prepared to perform an emergency cesarean section if indicated, and that the patient was consented prior to surgery for an emergency cesarean section for fetal indications [4,23]. Utilizing fetal monitoring during surgery can be difficult to interpret due to the lowering of baseline heart rate and minimal or absent variability of the fetal heart rate in the setting of anesthetic drugs. The main benefit of utilizing fetal heart rate monitoring intraoperatively would be to identify uteroplacental malperfusion and treat reversible causes. Overall, monitoring of fetal heart rate intraoperatively has not been associated with evidence of improved clinical outcomes [23].

Anesthetic techniques for non-obstetric surgery in pregnant patients can vary with reports showing the use of regional anesthesia, general anesthesia, or intravenous sedation. When choosing an anesthetic plan for the pregnant patient it is important to consider patient specifics, and the type of surgical procedure planned. Regional anesthesia is often the preferred anesthetic technique. However, literature has shown adverse outcomes when unusual anesthetic techniques are implored simply due to pregnancy. General anesthesia is the most common anesthetic technique utilized due to surgical needs. General anesthesia was shown to be associated with lower birth weight when compared to other anesthetic techniques in a 16-year retrospective cohort study, however, no differences were found in maternal or fetal mortality based on the type of anesthesia utilized [83].

Preferences for regional and neuraxial anesthesia are due to the maintenance of maternal respiratory drive and spontaneous ventilation, minimized fetal exposure to anesthetic drugs, and postoperative analgesia allowing decreased opioid consumption [15]. However, this modality of anesthesia is not without risk and those risks must be considered. Attention must be paid to the dosing of local anesthetic with proper confirmation of neuraxial placement due to the increased risk of local anesthetic toxicity from physiological changes of pregnancy increasing the unbound fraction of local anesthetic [84]. Given this risk of local anesthetic toxicity, lipid resuscitation should always be readily available for rapid initiation of treatment. Hypotension following neuraxial anesthesia is common and can lead to uteroplacental malperfusion. Therefore, it is paramount to maintain maternal blood pressure during and following neuraxial anesthetic placement with intravenous fluid bolus and resuscitation as well as with vasopressor medications, such as phenylephrine [23]. Shivering after neuraxial placement can ensue as well due to the blockade of thermoregulatory signaling. Maternal shivering can increase oxygen consumption and carbon dioxide production, both of which can impair placental perfusion [85].

Consideration should be given to ensure adequate motor and sensory blockade when neuraxial anesthesia is utilized. The decreased local anesthetic dosing helps decrease the risks previously discussed, however, it also decreases the time of blockade [85]. The anesthesiologist should consider adjuvants to the local anesthetic. The purpose of this is to improve the quality and duration of the anesthesia, as well as analgesia. Typical adjuvants include epinephrine, opioids (intrathecal morphine, fentanyl, or sufentanil), and alpha 2 adrenergic agonists (clonidine, dexmedetomidine). Opioid adjuvants are associated with pruritis, nausea and vomiting, hypotension, and delayed respiratory depression. Alpha 2 adrenergic receptor agonists are associated with dose-dependent bradycardia and hypotension [85]. However, the literature states when these adjuvants are used at recommended doses there are minimal fetal adverse effects. A mono-centric retrospective comparative study assessing Sufentanil vs. Dexmedetomidine as Neuraxial Adjuvants in Cesarean Section found that when comparing adjuvant use, sufentanil was associated with improved postoperative analgesia compared to dexmedetomidine. When assessing side effects, this study found that the most prominent side effect was pruritis in the sufentanil group and all other side effects were comparable in both groups. No difference was seen in fetal outcomes between groups [85].

However, when deciding on neuraxial versus general anesthesia for pregnant patients, the same considerations must be made to the contraindications of neuraxial anesthesia as when caring for nonpregnant patients. Absolute contraindications to neuraxial anesthesia include patient refusal, localized infection, increased intracranial pressure, and allergy to local anesthetic drugs. Relative contraindications include coagulopathy or use of anticoagulation drugs, preexisting neurologic impairment such as spinal stenosis, spinal bifida, or myelopathy, hypovolemia, and fixed cardiac outflow obstruction such as aortic stenosis [86].

General anesthesia is the most used anesthetic technique for non-obstetric surgery in pregnant patients, typically due to the needs of the planned surgical procedure. Due to physiologic changes during pregnancy, these patients will have a reduction in the functional residual capacity without a change in closing capacity in the setting of increased oxygen consumption, leading to rapid desaturation during apnea. Given this risk for apnea, effective preoxygenation with 100% fraction inspired oxygen to ideally an end-tidal oxygen percentage of >80% prior to induction of general anesthesia should be used [9,87]. Induction agents should be chosen in the same manner as non-pregnant patients as standard induction agents and dosing can and should be utilized. Regardless of the findings of low risk for pulmonary aspiration, most providers do elect to pursue RSI after 18 weeks gestation.

The neuromuscular blockade should be chosen based on plans for or against RSI and the need for continued paralysis throughout the surgical procedure as these drugs do not cross the placenta. Care should be taken with reversal of nondepolarizing neuromuscular blockade. It is known that sugammadex is a cyclodextrin molecule which binds to and encapsulates progesterone. Given progesterone’s critical role in the maintenance of pregnancy it is advised to avoid sugammadex in this population [88]. Therefore, neostigmine is commonly used for reversal of neuromuscular blockade in this patient population. A known consequence of neostigmine administration is bradycardia, which is why it is co-administered with glycopyrrolate. However, glycopyrrolate does not cross the placenta and there have been reports of fetal bradycardia without significant consequence [50]. Due to this, some suggest the administration of atropine with neostigmine as it more readily crosses the placenta to treat fetal bradycardia. Generally, glycopyrrolate is still used with neostigmine due to clinical comfort from the provider and lack of data showing consequences to the fetus [23].

Maintenance of anesthesia for surgery can be accomplished by either inhalational or intravenous anesthetic as both have been shown to be safe and effective for non-obstetric surgery in the pregnant patient. Considerations for the use of inhalation agents include the decreased minimum alveolar concentration in pregnancy, decreased uterine tone with use, and hypotension. When it comes to the use of nitrous oxide, there have been concerns that prolonged exposure can alter DNA synthesis through the inhibition of methionine synthetase. Animal studies have shown issues with teratogenicity when used during the period of peak organogenesis. No adverse outcomes have been shown in humans with short duration of exposure. However, most providers avoid use in the first trimester because of this [9,89]. For choice of analgesia during surgery, opioids have been shown to be safe. Ketamine has not been thoroughly studied. Ketamine use in pregnant patients is typically avoided due to high dosages causing increased tone and concern for fetal neurotoxicity [37].

Appropriate hemodynamic management intraoperatively is key to maintaining adequate uteroplacental perfusion and preventing maternal and fetal adverse outcomes. Uterine blood flow is not autoregulated and is reliant on maternal blood pressure. Anesthesia, regardless of neuraxial or general, leads to vasodilation and subsequently hypotension. It is recommended to maintain maternal blood pressure as close to baseline as possible with fluid resuscitation and vasopressor use if needed to preserve uteroplacental perfusion [23].

The goals of mechanical ventilation should consider the physiologic changes that are present in pregnancy, including increased minute ventilation with subsequent respiratory alkalosis. Lung protective strategies should still be employed with appropriate tidal volumes of 6-8cc/kg, respiratory rate can be increased to allow for increased minute ventilation. Positive end-expiratory pressure of at least 5 cm of water with up-titration as needed to help combat atelectasis. End-tidal carbon dioxide should be maintained around 30–32, as end-tidal carbon dioxide has been shown to be accurately reflective of arterial carbon dioxide. Extremes of carbon dioxide should be avoided as carbon dioxide easily crosses the placenta. Consequences of hypercarbia for the fetus include acidosis and myocardial depression, whereas hypocarbia can cause uteroplacental vasoconstriction and subsequent fetal hypoxia [90,91].

Temperature is another standard monitor put forth by the American Society of Anesthesiology. Use of a forced air warming blanket is recommended for procedures expected to exceed 60 min in duration. Normothermia should be maintained for pregnant patients as well as hypothermia can cause a decreased heart rate. The use of a forced air warming blanket is most effective, other adjuncts are warm blankets, fluid warmers, and increasing operating room temperature [92].

### 5.3. Postoperative Management

In the recovery area, the patient should continue to be in left uterine displacement to help prevent aortocaval compression until the patient is more wakeful and able to self-position. Monitoring in the recovery area should consist of standard monitoring with the addition of fetal monitoring based on previously discussed organizational guidelines. The presence of a qualified obstetrician during this monitoring is important to allow for interpretation of the monitoring and decide if tocolysis is indicated in the instance of preterm labor.

Analgesia in the recovery period is important, as poorly controlled pain produces maternal stress and can lead to adverse fetal outcomes [93]. Postoperative pain control can be accomplished in many ways, including local anesthetic wound infiltration by the surgical team, regional anesthesia, and non-opioid-based medications such as acetaminophen. Nonsteroidal anti-inflammatory drugs should be avoided after 20 weeks of pregnancy due to fetal consequences of renal dysfunction and oligohydramnios as stated in a release by the Federal Drug Administration [94]. In addition to the previously mentioned opioid-sparing modalities, the use of opioid-based medications for analgesia should not be withheld from the pregnant patient. These medications have not been shown to cause fetal adverse effects when used in the short term and should be provided to the pregnant patient in need of analgesia when indicated [37].

## 6. Conclusions

Non-obstetric surgery during pregnancy is a common occurrence and the pregnant patient undergoing this type of procedure can be managed safely with a multidisciplinary approach. It is never appropriate to delay a medically necessary surgery or procedure due to pregnancy as this is associated with worse maternal and fetal outcomes than performing the intervention. It is important to consider the physiologic changes that come with pregnancy to allow for appropriate anesthetic planning that prioritizes maternal as well as fetal well-being.

## Abbreviations

The following abbreviations are used in this manuscript:

ACOG	American College of Obstetrics and Gynecology
ASA	American Society of Anesthesiologists
FRC	Functional Reserve Capacity
FDA	Food and Drug Administration
RSI	Rapid sequence intubation
SCORE	Serious Complications Repository
